# Porphyria: What Is It and Who Should Be Evaluated?

**DOI:** 10.5041/RMMJ.10333

**Published:** 2018-04-19

**Authors:** Yonatan Edel, Rivka Mamet

**Affiliations:** 1 Porphyria Center, Rabin Medical Center, Beilinson Hospital, Petach Tikva, Israel; 2 Rheumatology Unit, Rabin Medical Center, Beilinson Hospital, Petach Tikva, Israel; 3 Sackler Faculty of Medicine, Tel Aviv University, Tel Aviv, Israel

**Keywords:** Aminolevulinic acid, porphobilinogen, porphyria

## Abstract

The porphyrias are a group of rare metabolic disorders, inherited or acquired, along the heme biosynthetic pathway, which could manifest with neurovisceral and/or cutaneous symptoms, depending on the defective enzyme. Neurovisceral porphyrias are characterized by acute attacks, in which excessive heme production is induced following exposure to a trigger. An acute attack usually presents with severe abdominal pain, vomiting, and tachycardia. Other symptoms which could appear include hypertension, hyponatremia, peripheral neuropathy, and mild mental symptoms. In severe attacks there could be severe symptoms including seizures and psychosis. If untreated, the attack might become very severe, affecting the peripheral, central, and autonomic nervous system, leading to paralysis, respiratory failure, hyponatremia, coma, and even death. From the biochemical point of view, acute attacks are involved with increased levels of precursors in the heme biosynthetic pathway, up to the deficient step. Of these precursors, aminolevulinic acid (ALA) is considered to be neurotoxic. Treatment is directed to reduce ALA production by reducing the activity of the enzyme aminolevulinate synthase (ALAS)—most effectively by heme therapy. Cutaneous symptoms are a consequence of elevated porphyrins in the blood stream. These porphyrins react to light; therefore sun-exposed areas are affected, producing fragile erosive skin lesions in porphyria cutanea tarda (PCT) or non-scarring stinging and burning symptoms in erythropoietic protoporphyria (EPP). Unlike the most common neurovisceral porphyria, acute intermittent porphyria (AIP), variegate porphyria (VP), and hereditary coproporphyria (HCP) can have cutaneous symptoms as well. Differentiating them from other cutaneous porphyrias is essential for accurate diagnosis, treatment, and patient recommendations.

## INTRODUCTION

**Figure 1:**
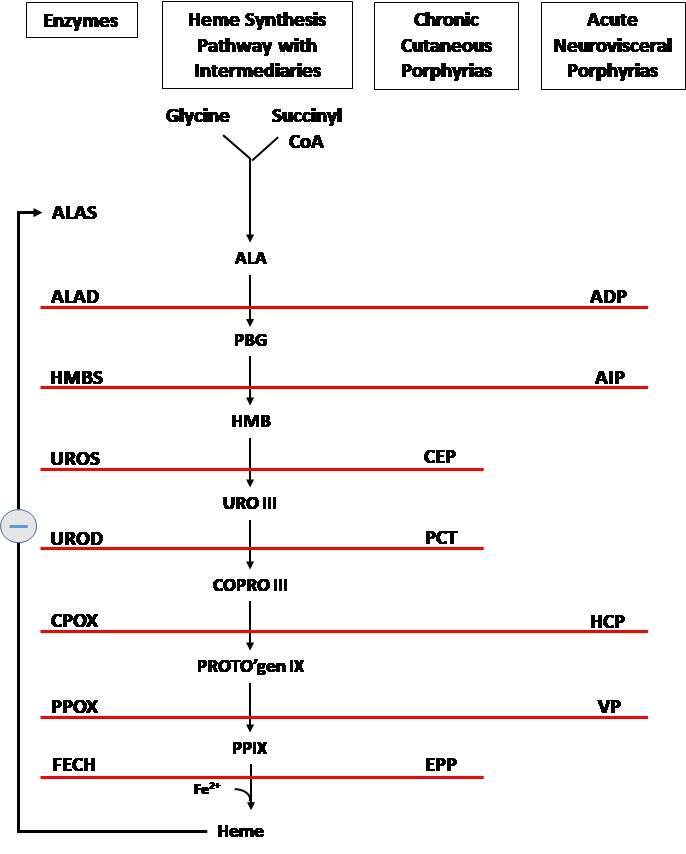
The Heme Synthesis Pathway: Enzymes Involved in the Pathway and the Associated Porphyrias with the Disruption of Each Specific Enzyme **Main (center) core**: Precursors and intermediary products in the heme synthesis pathway. (ALA, aminolevulinic acid; PBG, porphobilinogen; HMB, hydroxymethylbilane; URO III, uroporphyrinogen III; COPRO III, coproporphyrinogen III; PROTO’gen IX, protoporphyrinogen IX; PPIX, protoporphyrin IX; Fe2+, iron.) **Left of the core:** Enzymes, encoded by genes, catalyze each of the steps. Gene mutations cause deficient enzyme production. Disruptions are indicated by red lines connecting enzymes with the resultant porphyrias. (ALAS, aminolevulinate synthase; ALAD, aminolevulinic acid dehydratase; HMBS, hydroxymethylbilane synthase; UROS, uroporphyrinogen-III synthase; UROD, uroporphyrinogen decarboxylase; CPOX, coproporphyrinogen oxidase; PPOX, protoporphyrinogen oxidase; FECH, ferrochelatase.) **Right of the core:** Porphyrias resulting from disruption of enzyme production. (ADP, aminolevulinic acid dehydratase porphyria; AIP, acute intermittent porphyria; CEP, congenital erythropoietic porphyria; PCT, porphyria cutanea tarda; HCP, hereditary coproporphyria; VP, variegate porphyria; EPP, erythropoietic protoporphyria.) The final product, the heme, exerts control over the whole pathway via a negative feedback mechanism on the first enzyme – ALAS (indicated by the circle with the minus symbol).

The porphyrias are a group of rare metabolic disorders—either inherited or acquired along the heme biosynthetic pathway.^1^,^2^ Each type of porphyria is a result of a specific deficiency in one of the enzymes involved in the pathway (Figure 1) and, accordingly, is characterized by a specific pattern of accumulation of heme precursors and typical clinical manifestations.

Historically, porphyrias have been subdivided into hepatic and erythropoietic forms, according to the site of expression of the dysfunctional enzyme. However, in a clinical approach, it is much more convenient to classify them according to their clinical manifestations—as acute (neurovisceral) versus non-acute (cutaneous) porphyrias.^3^ In some of the acute porphyria types both neurovisceral and cutaneous symptoms may be present.

The acute neurovisceral forms are characterized by overproduction of delta-aminolevulinic acid (ALA) and porphobilinogen (PBG), which are porphyrin precursors, at the initial steps of heme synthesis^4^,^5^ (Figure 1), while the cutaneous ones are characterized by accumulation of porphyrins, which are the precursors at the final steps of the synthesis.^6^ This fundamental difference is the basis of the different clinical symptoms.

The accumulated precursors are excreted in the urine, in the feces, or in both—according to their solubility—and measuring their level is the basis of porphyria biochemical diagnosis and typing.^7^

Heme, the final product of the pathway, is essential for the synthesis of hematoproteins such as hemoglobin, myoglobin, microsomal cytochromes, catalase and others—all of which play an important role in oxygen transport and/or oxidation–reduction reactions. Most of heme synthesis in human beings (80%) takes place in erythropoietic cells, while about 15% is produced in the liver parenchymal cells. In both tissues, the synthesis is controlled, although differently.^3^

The first step in the synthesis, ALA formation (Figure 1), is the most important site of control. This step is catalyzed by the enzyme ALA synthase (ALAS), which has two subtypes—ALAS1, the ubiquitous one, encoded on chromosome 3, and ALAS2, erythroid-specific, encoded on chromosome X. In the erythropoietic tissue, regulation of heme synthesis is influenced by erythroid differentiation and erythropoietin and iron availability, while in the liver, ALAS1 is under negative feedback regulation by the intracellular heme pool.^3^ This regulatory mechanism is the basis of strategies in treatment and management of porphyria patients.

In this review we will summarize the different characteristics of each of the porphyrias: clinical manifestations, diagnosis, and treatment options.

## ACUTE PORPHYRIAS

The group of acute porphyrias includes four types of porphyria: acute intermittent porphyria (AIP), which is the most common one; hereditary coproporphyria (HCP); variegate porphyria (VP); and the rarest one, aminolevulinic acid dehydratase porphyria (ADP), of which only six definite cases have been reported so far and will be discussed later.

Two of these four diseases, HCP and VP, are associated with both acute attacks and skin bullous lesions,^8^ similar to those of non-acute cutaneous porphyrias, which will be discussed later in this review.

Acute intermittent porphyria (AIP), HCP, and VP are inherited in an autosomal dominant way and result from a deficiency of one of the enzymes, hydroxymethylbilane synthase (HMBS), coproporphyrinogen oxidase (CPOX), and protoporphyrinogen oxidase (PPOX), respectively (Figure 1). Since the deficiency is inherited from one affected parent, in all three porphyrias, the residual enzyme activity is about 50%, which is sufficient for regular heme homeostasis,^9^ keeping the disease latent. In fact, most patients will remain asymptomatic during their whole life without experiencing any porphyria symptoms.^10^

An acute attack usually occurs following an exposure to any one of the known precipitating factors. Most known precipitating factors are medications metabolized by the cytochrome P450 system—which are regarded as unsafe for porphyria patients.

Other factors include alcohol use, infections, low caloric intake, and changes in sex hormone balance during the menstrual cycle.^11^,^12^

Each of these precipitating factors induces ALAS1, either directly^13^,^14^ or indirectly by increasing demand for hepatic heme, mainly through the consumption of CYT P450 enzymes.^1^

Low carbohydrate intake might induce ALAS1 via peroxisome proliferator-activated receptor gamma coactivator 1-α (PGC-1α), a protein which directly induces transcription of ALAS1.^15^

When ALAS1 is up-regulated, the heme synthesis is accelerated and the deficient enzyme becomes a rate-limiting one. This leads to excess of heme precursors preceding the defective step.

Animal models^5^,^16^,^17^ and clinical evidence^4^ support the theory that delta aminolevulinic acid is neurotoxic, leading to the central, peripheral, and autonomic nervous system symptoms seen in an acute attack. In addition, some studies support the hypothesis suggesting that some of the symptoms of an acute attack might be mediated by increased serotonergic activity.^5^ Elevated levels of blood tryptophan and 5-OH tryptamine were reported in AIP patients,^18^ indicating abnormal tryptophan metabolism.

While, from the clinical point of view, acute porphyria attacks of all three types have similar symptoms and are indistinguishable, since they differ from each other in the defective enzyme, each subtype of porphyria has its own typical pattern of heme precursor excretion, which enables biochemical distinction between the three.^7^

The majority of acute attacks begin as a combination of abdominal pain, mild mental symptoms, such as severe fatigue and inability to concentrate, with or without autonomic dysfunction.^2^,^19^

Although all components of the peripheral, central, and autonomic nervous systems have been reported to be involved in an acute porphyria attack, the most common symptoms are severe abdominal pain, nausea, vomiting, and constipation. Tachycardia, hypertension, and signs of increased sympathetic activity are often associated with abdominal pain.^3^ Hyponatremia occurs in 40% of patients, probably as a result of inappropriate anti-diuretic hormone secretion syndrome. Additional factors that might contribute to hyponatremia include vomiting and resuscitation with high volumes of dextrose solutions given intravenously.^1^ Seizures, which are characteristic in severe attacks, may be due to severe hyponatremia, or, less commonly, due to posterior reversible encephalopathy syndrome (PRES).^20^

Liver enzymes might be mildly elevated during an attack.^19^ Severe attacks may also present with muscular weakness and or mental disturbance, such as anxiety, disorientation, or hallucinations.^1^,^2^ In rare cases of AIP, psychosis may be the only clinical manifestation.^21^,^22^

If a patient is not diagnosed early in the course of an attack, these symptoms can become very severe with full paralysis, respiratory failure, seizures, and even death.

Historically, the purple color of porphyrins, causing the dark colored urine in porphyria patients due to oxidation of PBG to uroporphyrin and porphobilin,^23^ gave the disease its name “porphyria.”

Commonly reported symptoms can be found in more detail in Table 1.

**Table 1:** Clinical Manifestations of Acute Attacks, with Symptoms Listed in the Order of Incidence In Each Box Separately.^19^

Clinical Manifestation	Symptoms in Order of Incidence
Autonomic Dysfunction	Abdominal pain Tachycardia Hypertension Constipation Vomiting and nausea Bladder paresis
Peripheral Neuropathy	Pain in the back and limbs Pareses/muscle weakness Low/absent tendon reflexes Respiratory paresis Cranial neuropathy Neuropathic sensory loss
Encephalopathy	Mild mental symptoms Seizures Coma Blurred vision Babinski signs Nystagmus Cerebellar ataxia
Metabolic Changes	Transaminases increased Pink/red/dark urine Hyponatremia

### Acute Porphyria Attack Diagnosis

The most rapid and accurate way to diagnose an acute porphyria attack is to measure urinary ALA and PBG levels, which are highly elevated during an acute attack. However, it should be pointed out that, in ADP porphyria, only urinary ALA and not PBG values will be increased.

**Figure 2:**
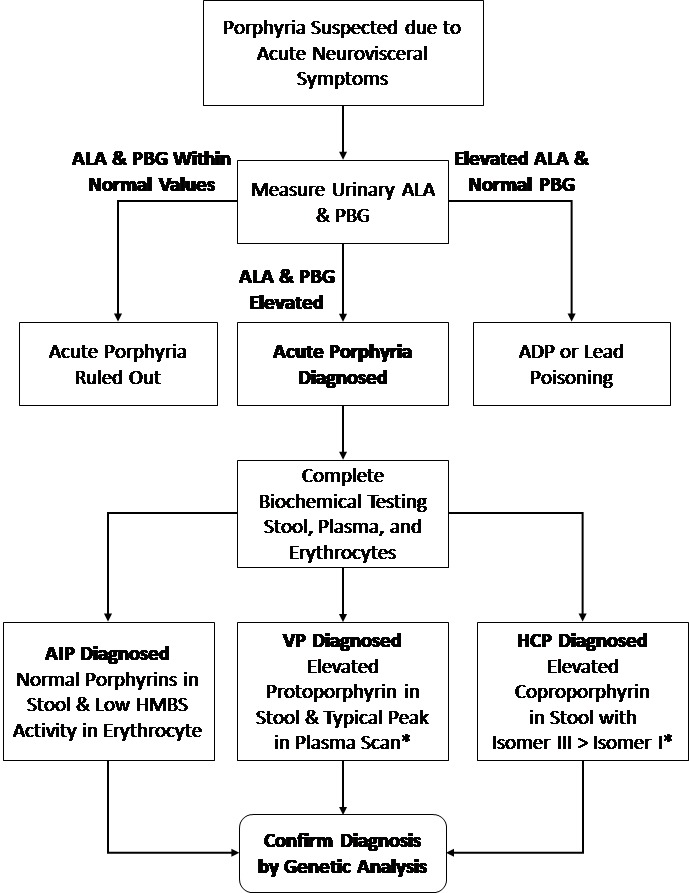
Diagnosis Algorithm for Acute Neurovisceral Symptoms Leading to Suspicion of Porphyria * Biochemical markers also detected in the latent phase. ADP, aminolevulinic acid dehydratase porphyria; AIP, acute intermittent porphyria; ALA, aminolevulinic acid; HCP, hereditary coproporphyria; HMBS, hydroxymethylbilane synthase; PBG, porphobilinogen; VP, variegate porphyria.

Once diagnosis has been made, then full biochemical evaluation, using stool, urine, and blood samples, is necessary to differentiate the type of acute porphyria affecting the patient (Figure 2).^7^

### Latent Acute Porphyria Diagnosis

Biochemical studies, performed on urine, feces, and blood, may identify each of the common acute porphyrias—AIP, HCP, and VP—even in silent patients.

Genetic testing is now available for all acute hepatic porphyrias following biochemical diagnosis, allowing family members to be screened for the specific mutation. Once a family member has been diagnosed with porphyria, he is considered a “latent patient” and should be advised to avoid all known manageable environmental factors which may trigger an attack.

At the time of writing, there have been over 400 different mutations identified only in AIP, and yet there is little evidence that a certain genotype could predict the phenotype.

### Acute Attack Treatment

As mentioned earlier, acute porphyria attacks may be life-threatening if not treated, due to severe neurological complications such as motor paralysis. Therefore, whenever an acute porphyria attack is diagnosed, the patient should be treated as early as possible.

The initial step of porphyria treatment should be to determine and withdraw any possible trigger.

Specific treatment, aimed at stopping the acceleration in heme synthesis which occurs during an acute attack, is targeted to down-regulate ALAS1 activity. This might be achieved, in very mild cases, by hydration and administration of carbohydrates, or much more effectively, in severe attacks, by blood-derived heme, such as Normosang (Orphan Europe, Puteaux, France) in Europe or Hematin (Xellia Pharmaceuticals, Nourth Carolina, USA) in the US.^2^

Palliative treatments given during an acute attack for pain, nausea, and other symptom relief should be performed using only “safe” drugs for porphyria patients. Data regarding safe drugs can be found on several web sites (including The Drug Database for Acute Porphyria at: www.drugs-porphyria.org).

Once an attack is over, a patient will usually gradually return to normal with no residual symptoms between attacks, unless exposure to precipitating factors is not withdrawn.

Some 3%–5% of newly diagnosed patients may suffer recurrent acute attacks.^10^,^24^ Management of these patients is challenging and, so far, has been based on one of the treatments below:

1. Heme therapy which could be given as needed, or in severe refractory patients given at regular intervals as prophylactic therapy.^25^ Complications of long-term heme infusions could include: iron overload, thrombotic superficial veins which could appear after transfusions, and dependence on the exogenic heme in some patients.^26^
2. In women suffering from menstrual cycle-related acute attacks, gonadotropin-releasing hormone (GnRH) agonist treatment could be given to avoid the attacks.^27^ Since these recurrent attacks tend to target young women, this treatment should be given only after considering other choices and discussing the physical and emotional issues carefully.
3. When no standard treatment is helpful and quality of life is poor, liver transplantation could be an option. Currently over 10 AIP patients have undergone liver transplantation since 2004.^10^,^26^ The potential of long- and short-term risks of liver transplantation and the prolonged immunosuppression leave this drastic option as a last resort.^10^

Lately, a subcutaneous therapy, based on RNAi targeted to silence ALAS1 expression (Givosiran, Alnylam Pharmaceuticals, Cambridge, MA, USA), has been developed and so far has shown preliminary promising results. This drug has recently been granted Food and Drug Administration (FDA) breakthrough therapy status.

Some patients are at risk for late complications. One of these complications includes chronic abdominal pain which is usually a late complication of recurrent acute attacks. It is important to differentiate this pain from an acute attack since it rarely responds to the same treatment.^28^

As for other possible complications, several studies suggest that patients with AIP are at higher risk for chronic kidney disease,^29^,^30^ with evidence showing this to be due to vascular toxicity of porphyrin metabolites to the tubular interstitium.^31^

There is also evidence showing an association between hepatocellular carcinoma and acute porphyria. This evidence has been mainly shown in northern European patients with AIP, although there are case reports in patients with VP and HCP^32^ too. Although the data are still lacking, currently, many porphyria services offer screening for porphyria patients over the age of 50.

### Epidemiology

As mentioned earlier, AIP is the most common acute hepatic porphyria affecting all ethnic and racial groups. In Europe the prevalence of symptomatic diagnosed AIP has been shown to be 5.9 per million.^24^ The actual prevalence of pathogenic mutations is unknown and thought to be much higher, with a clinical penetrance of only 1%–10%.^33^–^35^

Attacks occur more commonly in young females after puberty than in men and are rare after menopause.^29^ In AIP this female tendency is strongly observed, with up to 80%–90% of patients being female,^2^ while, in our experience, this is not as obvious in the case of other acute porphyrias.

Variegate porphyria (VP) is the second most common acute hepatic porphyria, and HCP is the third.^24^

While, as mentioned, all these acute porphyrias are inherited as an autosomal dominant disease, ADP is inherited in an autosomal recessive way, therefore being an extremely rare porphyria with only six certain reported cases confirmed by DNA studies.^36^ This type of porphyria results from a deficiency of the aminolevulinic acid dehydratase (ALAD) enzyme, resulting in a unique biochemical pattern during an acute attack with elevated urinary ALA level and a normal urinary PBG level (Figure 1).

### Homozygous Acute Porphyrias

Reported cases of homozygous AIP,^37^ HCP,^38^ and VP^39^ are rare. Homozygous AIP is associated with mental retardation, homozygous HCP with photosensitivity and neurological symptoms, and homozygous VP with severe photosensitivity and finger shortening.

### Lead Poisoning

An acquired ALAD inhibition, mimicking ADP, occurs in lead poisoning, in which symptoms may mimic those of acute porphyria.^40^,^41^ Treatment in this case should first address washing out the poisoning factor.

## CUTANEOUS PORPHYRIAS

### Porphyria Cutanea Tarda

Porphyria cutanea tarda (PCT) is the most common of the cutaneous porphyrias and is caused by a deficient level of uroporphyrinogen decarboxylase (UROD) (Figure 1). It can be subdivided into three types:

1. A sporadic acquired form constitutes 70%–80% of the cases. This sporadic form arises from known risk factors. These include: alcohol abuse, chronic hepatitis C, HIV infection, hemochromatosis, end-stage renal disease, and estrogen therapy. In the sporadic form, UROD activity is deficient in the liver, but not in erythrocytes.
2. A familial form constitutes about 20% of the cases and results from heterozygous mutation in one allele of UROD. The hereditary mutation reduces UROD activity in all tissues, but affected patients usually do not develop an overt disease unless exposed to additional risk factors.
3. A minority of the cases (less than 5%) have a positive family history in spite of normal erythrocyte UROD activity, implying that in these cases a genetic defect or common environmental factors remain unknown.^42^

Porphyria cutanea tarda is associated with cutaneous photosensitivity, and its clinical manifestations include blisters, bullae, or vesicular lesions restricted to sun-exposed areas (face, hands, forearms, and lower legs). The skin becomes very fragile, with superficial erosions covered by crust following minimal trauma.^43^

**Figure 3:**
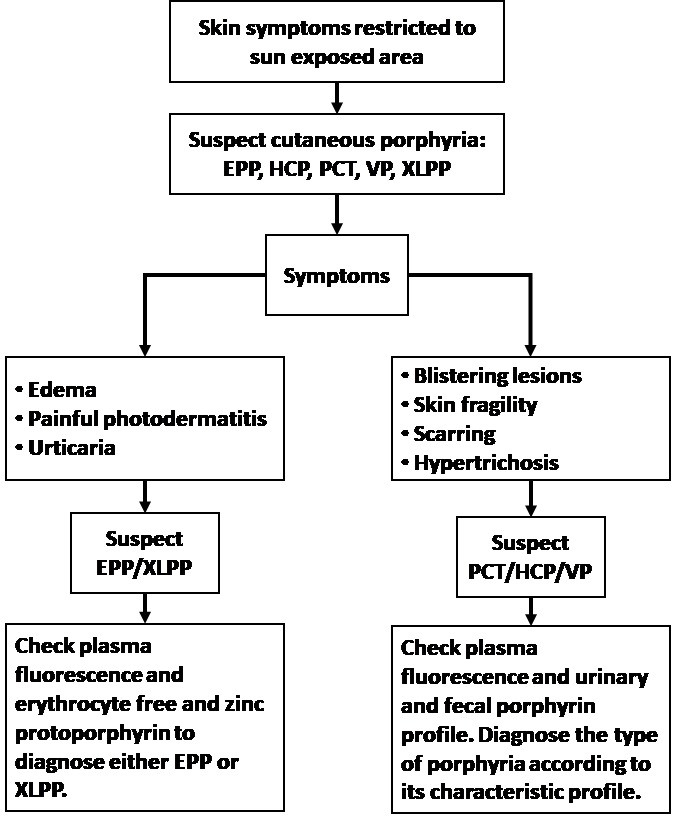
Diagnosis Algorithm for Porphyria Cutaneous Symptoms ADP, aminolevulinic acid dehydratase porphyria; AIP, acute intermittent porphyria; CEP, congenital erythropoietic porphyria; EPP, erythropoietic protoporphyria; HCP, hereditary coproporphyria; PCT, porphyria cutanea tarda; VP, variegate porphyria; XLPP, X-linked protoporphyria

While PCT has no neurovisceral manifestations, it is important to point out that patients with VP and HCP may display skin symptoms similar to those seen in PCT patients. Reliable biochemical testing to accurately diagnose the type of porphyria is essential, in order to avoid a possible acute attack of VP or HCP (Figure 3).

Homozygous familial PCT is extremely rare and is known as hepato-erythropoietic porphyria (HEP). This type of PCT is much more severe and develops during childhood, while the familial and sporadic forms appear at mid to late adulthood.^44^

### Treatment

Until plasma porphyrin levels normalize, patients should avoid direct sunlight.

The first therapeutic method should include treating and avoiding exposure to all the above-mentioned risk factors such as alcohol, smoking, estrogen, etc. In hepatitis C virus (HCV)-positive patients, antiviral treatment may cure both the hepatitis and the PCT.^43^

Besides controlling the risk factors, current direct treatments which are very effective in reducing porphyrin levels include: (1) iron reduction, either by phlebotomy, or, less effectively, by iron chelation; and (2) low-dose antimalarials, such as hydroxychloroquine or chloroquine, drugs that act as mobilizers of porphyrins from the liver, by transforming hepatocyte porphyrins into water-soluble complexes which excrete in the urine. Since initially there is an elevation in porphyrin levels some patients suffer initially from an increase in photosensitivity.

### Erythropoietic Protoporphyria

Erythropoietic protoporphyria (EPP) is a chronic erythropoietic porphyria, associated with excess accumulation of free protoporphyrin in erythroid cells, plasma, skin, and liver. Its clinical manifestation is mainly acute photosensitivity appearing as early as 20 minutes after exposure to light. This is a non-scarring reaction manifesting as pain, redness, and swelling of the exposed skin surface.^45^ Besides the cutaneous symptoms, patients often exhibit a slight microcytic, hypochromic anemia.^46^ A small percentage (approximately 2%) of EPP patients may develop hepatobiliary complications in addition to the cutaneous symptoms.

The genetic cause of the disease, in the majority of patients, is a partial deficiency in ferrochelatase, the last enzyme in the heme biosynthetic pathway.^47^ The inheritance of ferrochelatase deficiency EPP is complex and is usually associated with an inheritance of a mutated ferrochelatase (FECH) allele from one parent, and a low-expression allele (IVS-48T/C) from the other.^48^ Consequently, the residual activity of ferrochelatase is decreased below a critical threshold, leading to an overt disease of EPP. However, about 2%–10% of cases result from gain-of-function mutations in the ALAS2 gene. Since the ALAS2 gene is located on chromosome X, EPP associated with ALAS2 gain-of-function is inherited in a dominant X-linked way, due to which it is known as X-linked protoporphyria (XLPP). In female carriers, the phenotypic manifestation is directly influenced by the X-chromosomal inactivation.^49^

The prevalence of EPP worldwide is estimated to be 1:75,000 to 1:200,000, and is probably influenced by the frequency of IVS3-48C FECH allele in a given population.^50^

In Israel, the frequency of IVS3-48C FECH allele was investigated in the Ashkenazi population and was estimated to be similar to that found in European populations, 8%.^51^ Autosomal recessive inheritance, associated with mutations in both alleles of FECH, has been reported in about 4% of the cases.^52^ Patients with this recessive form of EPP usually exhibit palmar keratoderma.^53^

### Management

The main strategy in EPP management is avoiding exposure to light. Opaque sunscreen, barrier clothing, and protective tinted glass for cars and windows, is recommended. It is important to note that since skin symptoms are associated with exposure to light within the visible range, conventional sunblock creams are usually ineffective. Some products containing titanium dioxide might be of benefit.^54^,^55^ Some data have shown beta carotene therapy to be effective in reducing skin symptoms, although in a controlled study this effect has been shown to be minimal.^56^

Narrowband UVB phototherapy aimed to produce “skin hardening” may provide useful protection preventing cutaneous phototoxicity.^54^ Afamelanotide, a drug which has been recently developed (Clinuvel Inc., Melbourne, Australia), is an alpha-melanocyte-stimulating hormone analogue and is already in use, with good results.^57^

### Liver Failure

The proportion of EPP patients who develop severe liver complications is estimated to be about 5%.^2^ In these cases, removing protoporphyrin by exchange transfusions and plasmapheresis has been used as a bridge to liver transplantation. It is important to point out that, in surgical procedures, the use of protective filters for lights in the operating room is recommended, in order to avoid phototoxic damage to internal organs.^55^ Immunization for hepatitis A and B is recommended as well.^55^

In all types of cutaneous porphyria, avoidance of sunlight might contribute to vitamin D deficiency^58^,^59^ and lower bone density.^60^ It is highly recommended to monitor serum vitamin D status and treat with supplementations if needed.

### Congenital Erythropoietic Porphyria

Congenital erythropoietic porphyria (CEP), also known as Günther’s disease, is an extremely rare autosomal recessive cutaneous porphyria**,** with a prevalence of less than 1 per million. This kind of porphyria is caused by deficient activity of uroporphyrinogen-III synthase (UROS), the fourth enzyme in the heme biochemical pathway. The clinical manifestations start in early infancy and may affect different organs. They include: skin fragility, blistering, and scarring; facial hypertrichosis and/or scarring alopecia; hemolytic anemia or pancytopenia; scarring of fingers, resorption of finger tips; photophobia and loss of eye lashes; erythrodontia; neonatal jaundice; and splenomegaly.^61^

Treatment for CEP starts with sun protection to avoid skin trauma. Since the symptoms may involve several systems, the treatment depends on the affected organ. So far, the only successful curative strategy reported is bone marrow transplantation.^58^

## CONCLUSION

The porphyrias are a group of diseases that may exhibit various symptoms, according to the type of disease. Consequently, physicians from many disciplines may meet undiagnosed porphyria patients of any type. Awareness of porphyria acute neurovisceral attacks or cutaneous symptoms may lead to an accurate diagnosis and, as a result, to prompt treatment, which might be life-saving.

## Abbreviations

ADP: aminolevulinic acid dehydratase porphyria
AIP: acute intermittent porphyria
ALA: aminolevulinic acid
ALAD: aminolevulinic acid dehydratase
ALAS: aminolevulinate synthase
CEP: congenital erythropoietic porphyria
COPRO III: coproporphyrinogen III
CPOX: coproporphyrinogen oxidase
EPP: erythropoietic protoporphyria
Fe^2+^: iron
FECH: ferrochelatase
HCP: hereditary coproporphyria
HEP: hepato-erythropoietic porphyria
HMB: hydroxymethylbilane
HMBS: hydroxymethylbilane synthase
PBG: porphobilinogen
PCT: porphyria cutanea tarda
PPOX: protoporphyrinogen oxidase
PROTO’gen IX: protoporphyrinogen IX
URO III: uroporphyrinogen III
UROD: uroporphyrinogen decarboxylase
UROS: uroporphyrinogen-III synthase
VP: variegate porphyria
XLPP: X-linked protoporphyria.
